# SARS-CoV-2 seroprevalence among Beninese pregnant women in the third year of the pandemic

**DOI:** 10.1186/s12889-024-19087-4

**Published:** 2024-07-02

**Authors:** Antía Figueroa-Romero, Aurore Atchadé, Anges Yadouleton, Marc Fiogbe, Emmanuel Bonnet, Emmanuel Yovo, Manfred Accrombessi, Sandrine Hounsa, Thierry Paper, Raphael Dupont, Jean Gaudart, Jean-Yves Le Hesran, Achille Massougbodji, Gilles Cottrell, Raquel González

**Affiliations:** 1 Barcelona Institute for Global Health (ISGlobal), Hospital Clínic- Universitat de Barcelona, Barcelona, Spain; 2 Consorcio de Investigación Biomédica en Red de Epidemiología y Salud Pública, CIBERESP, Madrid, Spain; 3 Institut de Recherche Clinique du Bénin, Abomey-Calavi, Benin; 4 Laboratoire des fièvres hémorragiques virales du Bénin, Ministère de la Santé du Bénin, Cotonou, 01BP918 Bénin; 5 Institut de recherche pour le développement PRODIG UMR 215, CNRS Université Paris 1 Panthéon- Sorbonne, AgroParisTech 5, cours des Humanités, Aubervilliers, Île-de-France, F-93 322 France; 6 Faculty of Infectious and Tropical Diseases, Disease Control Department, London School of Hygiene & Tropical Medicine, London, UK; 7 Population Services International, Malaria Department, Country-Based Global Employee, Cotonou, Benin; 8 Biosynex S.A, 22 boulevard Sebastien Brant, Illkirch Graffenstaden, Strasbourg, F-67400 France; 9 Aix Marseille Univ, IRD, INSERM, SESSTIM, ISSPAM, AP-HM, Hop La Timone, BioSTIC, Biostatistic and ICT, Marseille, France; 10 Institut de Recherche pour le Développement, MERIT UMR216, Université Paris-Cité, Faculté de pharmacie, laboratoire de parasitologie, Paris, France; 11 Department of Medicine, Faculty of Medicine, Universitat de Barcelona, Barcelona, Spain; 12 Centro de Investigação em Saúde de Manhiça, Maputo, Mozambique

**Keywords:** Pregnancy, SARS-CoV-2, Sub-saharan Africa

## Abstract

**Background:**

Pregnant women are a vulnerable population to COVID-19 given an increased susceptibility to severe SARS-CoV-2 infection and pregnancy complications. However, few SARS-CoV-2 serological surveys have been performed among this population to assess the extent of the infection in sub-Saharan countries. The objectives of this study were to determine SARS-CoV-2 seroprevalence among Beninese pregnant women, to identify spatial seropositivity clusters and to analyse factors associated with the infection.

**Methods:**

A cross-sectional study including women in their third trimester of pregnancy attending the antenatal care (ANC) clinics at Allada (south Benin) and Natitingou (north Benin) was conducted. Rapid diagnostic tests (RDT) for detection of IgG/IgM against the SARS-CoV-2 spike protein were performed using capillary blood. Seroprevalence of SARS-CoV-2 antibodies and associations between SARS-CoV-2 serostatus and maternal characteristics were analyzed by multivariate logistic regression. Spatial analyses were performed using the spatial scan statistics to identify spatial clusters of SARS-CoV-2 infection.

**Results:**

A total of 861 pregnant women were enrolled between May 4 and June 29, 2022. 58/861 (6.7%) participants reported having received COVID-19 vaccine. None of the participants had been diagnosed with COVID-19 during their pregnancy. SARS-CoV-2 antibodies were detected in 607/802 (75.7%; 95% CI 72.56%–78.62%) of unvaccinated participants. Several urban and rural spatial clusters of SARS-CoV-2 cases were identified in Allada and one urban spatial cluster was identified in Natitingou. Unvaccinated participants from Allada with at least one previous morbidity were at a three-times higher risk of presenting SARS-CoV-2 antibodies (OR = 2.89; 95%CI 1.19%-7.00%).

**Conclusion:**

Three out of four pregnant women had SARS-CoV-2 antibodies, suggesting a high virus circulation among pregnant women in Benin, while COVID-19 vaccination coverage was low. Pregnant women with comorbidities may be at increased risk of SARS-CoV-2 infection. This population should be prioritized for COVID-19 diagnosis and vaccination in order to prevent its deleterious effects.

**Trial registration:**

NCT06170320 (retrospectively registered on December 21, 2023).

## Background

In Africa, as of April 2024, more than 9.5 million cases of coronavirus disease (COVID-19) have been reported, representing 1.2% of the cases globally [1]. The first COVID-19 case in Benin was reported on March 16, 2020 [1]. As of April 2024, 28,036 cases and 163 deaths of COVID-19 have been notified [1]. However, the true extent of the pandemic may be much higher than officially reported. Serological surveys performed in Benin, in March and May 2021 in Cotonou and in August 2021 in Natitingou, found seroprevalences of severe acute respiratory syndrome coronavirus 2 (SARS-CoV-2) of 29.8% (Cotonou, March), 34.9% (Cotonou, May) and 33.3% (Natitingou, August) [2].

Pregnant women are vulnerable to COVID-19 given an increased susceptibility to suffering severe COVID-19, mostly explained by pregnancy-associated physiologic changes [3]. These include a decreased lung volume, an increased risk for thromboembolic disease and some pregnancy-related immunological alterations [3]. In addition, pregnant women are at increased risk of severe pregnancy complications such as preterm birth, preeclampsia and maternal death [4–6].

The World Health Organization (WHO) recommended that all countries carry out population-based SARS-CoV-2 seroprevalence surveys to measure the seroprevalence of antibodies to SARS-CoV-2 and estimate the proportion of symptomatic and asymptomatic populations [7]. However, as of April 2024, only eight studies had assessed the SARS-CoV-2 seroprevalence among pregnant women in the African continent, only one in West Africa [8, 9]. In Egypt, from July to September 2020, the prevalence of IgG was 25% among asymptomatic at low-risk pregnant women [10]. In East Africa, a serological survey in Ethiopia from April 2020 to March 2021 found a cumulative prevalence of 5.7% [11]. Two surveys performed in different sites in Kenya found a prevalence of 33.1% in Kafue and Chongwe in October 2021 [12], and a prevalence of 63% and 82% in Kilifi County and Nairobi, respectively, in the same period [13]. This same study, which assessed SARS-CoV-2 seroprevalence in other sub-Saharan African countries, found a seroprevalence of 31.7% and 37.8% in the Democratic Republic of Congo and Zambia, respectively, in October 2021 [12]. In the capital city of Somalia, the seroprevalence of SARS-CoV-2 antibodies was 36.5% between July and August 2021 [14]. In Southern Africa, a study performed in southern Mozambique between November 2019 and June 2021 found an overall seroprevalence of 11.3% among HIV-infected unvaccinated pregnant women [15]. In Johannesburg (South Africa) the seroprevalence of SARS-CoV-2 between March and June 2021 was 64.0% among unvaccinated pregnant women [16]. SARS-CoV-2 seroprevalence in The Gambia ranged from 0% before the onset of the pandemic to 90% in December 2021 [9]. The heterogeneity in the results among studies may be due to the timing of the studies, chance variation, differences in health infrastructures and mitigation efforts, or the type of diagnostic test used [17]. Routine surveillance systems in countries within the WHO African region, including Benin, may have underestimated COVID-19 cases. Understanding the true extent of infection, its geographical distribution and risk factors in pregnancy is fundamental for the allocation of scarce health resources, including vaccines [18]. In this study, we aimed to evaluate the seroprevalence of the SARS-CoV-2 infection and identify factors associated with seropositivity as well as spatial clusters of SARS-CoV-2 infection among pregnant women in two Beninese settings.

## Methods

### Study area

The study was conducted in two cities of Benin: Allada and Natitingou. Allada is a semi-rural area located 50 km north of Cotonou, with an estimated population of 127,512 in 2013. Natitingou is a city located more than 500 km north of Cotonou near the Burkina Faso border. Natitingou had an estimated population of 128,511 in 2020. Three antenatal care (ANC) clinics were purposively selected to be included in the study: *Maternité de l’Hôpital de Zone d’Allada, Maternité de Centre du Santé Communale d’Allada,* and *Maternité de l’Hôpital de Zone Mère et Enfant de Natitingou.* The selection of the study ANC clinics was done in collaboration with the Beninese Ministry of Health, and based on the clinics’ expected influx of pregnant women.

### Study design and procedures

This is a cross-sectional study to determine the seroprevalence of SARS-CoV-2 antibodies in pregnant women. All women attending the ANC clinic during their third trimester (≥ 28 weeks) of pregnancy were invited to participate. The rationale behind focusing on pregnant women in their third trimester is to increase the likelihood of capturing participants who were infected during their pregnancy.

For sample size calculation, we used the 30% SARS-CoV-2 seroprevalence estimate from the general population-based results from a study conducted in Benin in 2021 [2]. A sample size of at least 323 women was calculated to be needed to determine the prevalence of infection with a precision of 0.05 and a 95% confidence interval (95% CI).

SARS-CoV-2 seroprevalence was defined as the proportion of participants presenting antibodies in blood against the SARS-CoV-2 spike (S) protein. An electronic case report form was administered to record women’s demographic and clinical data, including the participants’ neighbourhood of residence. The information was self-reported, except for information regarding COVID-19 vaccination, which was cross-checked with the participants’ vaccination card. A RDT (COVID-19 self-test, BIOSYNEX Swiss SA, Freiburg, Switzerland) lateral-flow immunochromatographic assay was performed for qualitative detection of IgG/IgM against the receptor binding domain of SARS-CoV-2 S protein, with a reported a sensitivity of 100.0% (95%CI 90.5%-100.0%) and specificity of 100% (95%CI 96.3%-100.0%) using an ELISA kit performed in the general population as the comparator method (personal communication).

### Statistical analysis

The primary study outcome was the prevalence of total antibodies (defined as the presence of IgG and/or IgM) against SARS-CoV-2 detected in capillary blood samples. Seroprevalence of Ig against SARS-CoV-2 was estimated as proportions with 95% CIs. The participants’ socio-demographic and clinical characteristics at baseline are presented as proportions (for categorical variables) and mean and standard deviation (for quantitative variables). Bivariate logistic regression was used to test associations between the presence of SARS-CoV-2 antibodies and maternal characteristics and to estimate odds ratio (ORs) and 95 CIs. Multivariate analysis was also performed by logistic regression and adjusting for variables selected based on their association in the univariate analysis with a *p*-value < 0.2 or based on the literature (age, education and employment status). These are the standard recommended statistical test to assess associations between qualitative variables [19, 20]. Malnutrition was defined as having a middle upper arm circumference (MUAC) ≤ 23 centimetres (cm) following current SPHERE guidelines and literature [21, 22].

### Geospatial analysis

We used Kulldorff’s scan spatial statistic to detect spatial clusters of COVID-19 cases. Scan statistics detect geographical areas with higher-than-expected disease incidence in order to identify the sources of an epidemic and to verify whether the geographical clustering was due to random variation. The window with the maximum likelihood was defined as the most likely cluster area, and other clusters with statistically significant log likelihood ratios (LLRs) were defined as potential secondary clusters. LLR *p*-values were estimated by 9999 Monte Carlo simulations. A *p*-value < 0.05 indicates a significantly elevated risk within the analysis window, which could be a potential high-risk cluster for COVID-19. The relative risk (RR) of COVID-19 in each cluster was calculated to assess the risk of COVID-19 in the cluster zones. The spatial analyses were performed using spatial scan statistics implemented in SatScan (version 9.4) [23, 24]. This method detects regions of higher-than-expected disease incidence in time and space by assigning them a relative risk, producing a list of spatial clusters that can be used to identify the epidemic concentration in the study area.

## Results

### Characteristics of study participants

A total of 861 women were enrolled in the study between May 4 and June 29, 2022; of them, 455 participants were recruited in Allada and 406 in Natitingou.

Socio-demographic and clinical characteristics of participants are displayed in Table 1. More than half of the participants (54.1% and 58.6% in Allada and Natitingou, respectively) were older than 25 years (age range: 13–45). The mean gestational age was 35.7 and 34.2 weeks in Allada and Natitingou, respectively. Only 7.3% of participants from Allada and 6.2% of participants from Natitingou had been vaccinated against COVID-19. In total, 13.7% of participants presented pre-existent comorbidities (at least one of obesity, diabetes, HIV, hypertension, asthma or other pulmonary pathology, or malnutrition) in Allada and 9.9% of participants in Natitingou.

**Table 1:** Participants’ sociodemographic and clinical characteristics

	Allada [*N* = 455]	Natitingou [*N* = 406]	Total
Age (years)			
< 25 ≥ 25	209 (45.9) 246 (54.1)	168 (41.4) 238 (58.6)	377 (43.8) 484 (56.2)
Gravidity			
Primigravidae Multigravidae	61 (13.4) 394 (86.6)	90 (22.2) 316 (77.8)	151 (17.5) 710 (82.5)
Gestational age (weeks) ^1^	35.7 (3.8)	34.2 (4.1)	35.0 (4.0)
Educational level			
None Primary Secondary or higher	196 (43.1) 127 (21.9) 132 (29.0)	99 (24.4) 43 (10.6) 264 (65.0)	295 (34.3) 170 (19.7) 396 (46.0)
Employment			
Unemployed Employed	20 (4.4) 435 (95.6)	38 (9.4) 368 (90.6)	58 (6.7) 803 (93.3)
Marital status			
Single Married	3 (0.7) 452 (99.3)	14 (3.5) 392 (96.5)	17 (2.0) 844 (98.0)
COVID-19 vaccination			
Vaccinated* Unvaccinated Do not know	33 (7.3) 422 (92.7) 0 (0.0)	25 (6.2) 380 (93.6) 1 (0.2)	58 (6.7) 802 (93.2) 1 (0.1)
Pre-existent morbidities**			
Yes No	62 (13.7) 392 (86.3)	40 (9.9) 364 (90.1)	102 (11.9) 756 (88.1)

Values are number n (%) unless indicated otherwise

^1^mean (SD)

*At least one COVID-19 vaccine

**Obesity, diabetes, HIV, hypertension, asthma or other pulmonary pathology, malnutrition.

### Seroprevalence of anti-SARS-CoV-2 antibodies

SARS-CoV-2 seroprevalence by site and vaccination status is displayed in Table 2. A total of 658 (76.4%, 95% CI 73.44%-79.22%) participants tested positive for SARS-CoV-2 antibodies. In Allada, 352 (77.4%, 95% CI 73.23%-81.13%) presented SARS-CoV-2 antibodies, while in Natitingou, 306 (75.4%, 95% CI 70.87%-79.49%) participants tested positive. According to vaccination status, 607 (75.7%, 95% CI 72.56%-78.62%) unvaccinated participants presented SARS-CoV-2 antibodies. Overall the proportion of participants that presented SARS-CoV-2 antibodies when fully vaccinated was 87.9% (95%CI 76.70%-95.01%).

**Table 2:** SARS-CoV-2 seroprevalence in Allada and Natitingou by COVID-19 vaccination status

	Allada *n*/*N* (%)	Natitingou *n*/*N* (%)	Both sites *n*/*N* (%)
Unvaccinated	323/422 (76.5)	284/380 (74.7)	607/802 (75.7)
Vaccinated	29/33 (87.9)	22/25 (88.0)	51/58 (87.9)
All participants*	352/455 (77.4)	306/406 (75.4)	658/861 (76.4)

*One participant reported not knowing whether she had been vaccinated; thus, she is only considered in the “all participants” category

No study participant reported a history of positive COVID-19 diagnosis during their pregnancy in our study, nor having been in close contact with a positive SARS-CoV-2 case.

A verification study of the RDT used, performed in 437 samples from the general population, returned a sensitivity of 97.2% and specificity of 68.7% using enzyme-linked immunosorbent assay (ELISA) as the gold standard. This ELISA test detected IgG against the SARS-CoV-2 S protein. The positive predictive value (PPV) and the negative predictive value (NPV) were 93.0% and 85.1%, respectively.

### Maternal factors associated with anti-SARS-CoV-2 seropositivity

Having at least one pre-existing morbidity was associated with a statistically significant increased risk of presenting SARS-CoV-2 antibodies in the multivariate analysis (OR = 1.95, 95%CI 1.07%–3.54%) (Table 3). This significance was kept when the analysis was performed among participants from Allada (adjusted OR = 2.89 [95%CI 1.19%-7.00%]), but not in participants from Natitingou (adjusted OR = 1.28, 95%CI 0.56%–2.94%). No pregnancy complication (pre-eclampsia, gestational diabetes or threatened preterm birth) was found to be associated with SARS-CoV-2 seropositivity either in Allada or in Natitingou.

**Table 3:** Multivariate analysis of factors associated to presence of SARS-CoV-2 antibodies among unvaccinated participants by study site and combined

	Allada		Natitingou		Both sites combined	
	Adjusted OR (95CI)	Adjusted *p*-value	Adjusted OR (95CI)	Adjusted *p*-value	Adjusted OR (95CI)	Adjusted *p*-value
Age (years) < 25 ≥ 25	Ref 1.53 (0.97–2.44)	0.068	Ref 0.95 (0. 58-0.56)	0.852	Ref 1.24 (0.89–1.73)	0.200
Education None Primary Secondary or higher	Ref 1.18 (0.68–2.05) 1.18 (0.66–2.08)	0.780	Ref 1.33 (0.57–3.23) 1.10 (0.66–1.94)	0.770	Ref 1.21 (0.77–1.91)	0.705
Employment Employed Unemployed	Ref 3.19 (0.71–14.37)	0.130	Ref 1.06 (0.41–2.54)	0.901	Ref 1.53 (0.73–3.17)	0.258
At least one previous morbidity^†^ No Yes	Ref 2.89 (1.19-7.00)	0.019	Ref 1.28 (0.56–2.94)	0.553	Ref 1.95 (1.07–3.54)	0.028

OR: Odds ratio; Ref: reference; 95CI: 95% Confidence Interval

^†^At least one of obesity, diabetes, VIH/other immunodeficiency, hypertension, asthma or other pulmonary pathology, or malnutrition

### Geographical distribution of SARS-CoV-2 seropositive pregnant women

**Fig. 1:**
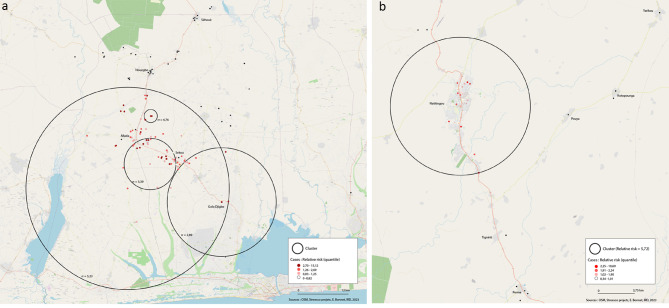
(**a**) Significant spatial clusters in Allada, (**b**) Significant spatial clusters in Natitingou

A significant spatial cluster of SARS-CoV-2 seropositivity was found in Allada and Natitingou (Table 4). Four significant clusters of 23.15, 5.88, 12.42 and 1.50 km radius were identified in Allada (Fig. 1a), covering rural and urban areas, while one cluster of a 7.48 km radius was identified covering the urban area of Natitingou (Fig. 1b).

**Table 4:** Statistically significant spatial clusters of SARS-CoV-2 cases detected

Site	Coordinates	Radius (km)	Relative risk	*P*-value
Allada	6.572163 N, 2.129683 E	23.15	5.24	< 0.001
	6.622464 N, 2.175918 E	5.88	3.39	< 0.001
	6.544191 N, 2.323700 E	12.42	2.89	< 0.001
	6.721236 N, 2.177389 E	1.50	4.77	< 0.001
Natitingou	10.294537 N, 1.380047 E	7.48	5.73	< 0.001

## Discussion

This study is among the first to assess SARS-CoV-2 seroprevalence among pregnant women in West Africa, as well as its associated risk factors and its geographical distribution. In this cross-sectional study performed between May 4 and June 29, 2022, only 6.7% of the participants reported previous COVID-19 vaccination. Among the unvaccinated, 75.7% participants presented SARS-CoV-2 antibodies. Unvaccinated participants from Allada who had at least one previous morbidity had a three-fold increased risk of presenting SARS-CoV-2 antibodies. None of the participants had tested positive for COVID-19 during their pregnancy. There was spatial clustering of SARS-CoV-2 seropositivity in urban and rural zones from Allada and in the urban area of Natitingou.

In Benin as of April 2024, 28,036 cases of COVID-19 had been reported [1]. None of the study participants had been diagnosed with COVID-19 during their pregnancy, and although there is no data on prior COVID-19 diagnosis, it is most likely that COVID-19 cases may have not been entirely captured by routine surveillance systems, as 76.4% of the unvaccinated study participants presented antibodies, which is also in accordance with the seroprevalence studies performed in the general population in Cotonou and Natitingou [2]. In the same line, a meta-analysis reporting on SARS-CoV-2 global seroprevalence has found that the ratio of seroprevalence to cumulative incidence of confirmed cases reported by the WHO ranged from 82.2:1 in July-September 2020 to 176.7:1 in July-September 2021 in the WHO African region, in comparison to global ratios ranging from 51.3:1 to 10.5:1. This suggests that routine surveillance systems from countries in the WHO African region have highly underestimated COVID-19 cases [10], and highlights the importance of performing serological studies to assess the true extent of the pandemic.

The underreporting of COVID-19 cases in Benin has already been pointed by SARS-CoV-2 seroprevalence surveys performed at the community level in the country, which found that SARS-CoV-2 seroprevalence was over 30% from March to August 2021 [2]. In addition, our results, obtained one year afterwards, suggest that SARS-CoV-2 seroprevalence has raised greatly from mid-2021 to mid-2022. It is thus likely that the virus has circulated extensively in the country during that period, which corresponds with the emergence of the SARS-CoV-2 Omicron variant. This variant was first identified in South Africa in November 2021 and soon spread globally. Similar patterns of rapid growth have been reported in other countries across the world [25, 26], and this high seroprevalence after the omicron wave is in accordance with other studies performed inside and outside West Africa during this period [8, 27–30].

Our data indicates that pregnant women who had at least one comorbidity were at a statistically significant two times-higher risk of presenting SARS-CoV-2 antibodies than those with no comorbidities. When stratifying by study area, this significance was maintained in participants from Allada. Studies performed in Africa among pregnant women and other population groups found that individuals with comorbidities were at a higher risk of presenting SARS-CoV-2 antibodies [11, 14, 31–33]. These individuals are more likely to suffer more severe COVID-19 symptoms [34], while severe illness is linked to stronger and longer-lasting responses [35]. Thus, serological surveys may detect antibodies more frequently in people with comorbidities. However, these results were not replicated in participants from Natitingou. Similarly, other studies also failed to find an association between SARS-CoV-2 seropositivity and presence of comorbidities [36–39]. In this sense, there are still gaps in the understanding of antibody levels and duration of protection against SARS-CoV-2 in people with comorbidities.

The area of residence has been reported as a factor that may influence SARS-CoV-2 acquisition [40, 41]. People living in urban areas are frequently reported as being at higher risk of contracting the disease [40, 41], which is consistent with the present results showing a seropositivity hotspot covering the whole urban area of Natitingou. In contrast, the findings from the spatial analysis in Allada indicate that there was not any specific pattern in the distribution of SARS-CoV-2 infection in terms of area of residence among pregnant women given that several significant clusters were identified, including both rural and urban areas. This may be explained by the fact that despite people living in urban areas may have been more exposed to SARS-CoV-2, people living in rural areas, including pregnant women, may have faced challenges such as less access to preventive measures and information, which in turn lead to a poorer health literacy and may have hampered their ability to prevent the disease [42].

Because pregnant women are an accessible population given their frequent contact with the health system, they have traditionally been proposed as a sentinel population group to monitor trends of the prevalence of different infectious diseases in the community such as HIV and malaria [43, 44]. According to our results, pregnant women are likely to present seroprevalence levels and dynamics similar to those of the general population for SARS-CoV-2 infection and thus may be considered as a sentinel population for SARS-CoV-2 surveillance, and other potential epidemics, which has already been suggested by other studies [9, 11, 45–48]. However, caution should be taken, given that pregnant women may present unique characteristics such as behaviours to increase their protection that may limit its suitability [46, 47].

Despite pregnant women are at increased risk of severe COVID-19 and pregnancy complications [4, 5], only 6.7% of the pregnant women included in this study reported having received at least one COVID-19 vaccine dose in comparison to 23.7% of the Beninese population that had been vaccinated with at least two doses as of June 2022, when data collection was completed [1, 49]. This may reflect the fact that COVID-19 vaccination in pregnancy was not recommended in many low-income countries when it was first deployed [50], and even after their use was permitted, pregnant women seemed to be a population particularly reluctant to get vaccinated [51–53]. Reasons for this hesitancy include concerns regarding the safety of COVID-19 vaccination during pregnancy and its side effects [51–53]. A study performed in Kenya among pregnant women also found that the proportion of participants vaccinated with at least one dose in Nairobi (13%) was lower than the average for adults at that time (October 2021, 34%) [13]. Despite the implementation of the COVAX initiative, vaccine coverage was far from the global target of 70% by the end of 2022 [18], and even further for Beninese pregnant women. Focusing COVID-19 immunization efforts on vulnerable populations where the greatest burden of the disease concentrates may be a suitable strategy to tackle the disease [54]. This includes addressing vaccine hesitancy through strategies to reinforce perceptions of COVID-19 vaccine safety, such as interpersonal and mass media channels, and engaging the community to support pregnant women in the decision to accept vaccination against COVID-19 [55].

Our study is constrained by the specificity of the RDT used. As with any other laboratory test, it may present measurement error, which could result in a biased prevalence estimate. This specificity may be due to the fact that the ELISA test used as a gold standard did not detect anti-SARS-CoV-2 IgM, thus, it may have underestimated the true proportion of positive samples. In addition to this, the sensibility, PPV and NPV were high. Moreover, the RDT used for antibodies diagnosis does not differentiate anti-S IgG and IgM, thus this prevent us from assessing recent versus past infection. In addition, the presence of antibodies in the study participants does not prove with certainty that the infection occurred during their pregnancy, given that seroconversion of anti-S IgG antibodies is highly heterogeneous among subjects (half-life ranges 89 to 325 days) [56]. However, in order to increase the probability of the infection taking place during their pregnancy, participants were recruited during their third semester of pregnancy. Finally, the information regarding participants’ comorbidities was self-reported; thus, the results derived from the multivariate analysis should be interpreted with caution.

## Conclusions

In conclusion, the seroprevalence of SARS-CoV-2 among pregnant women from Allada and Natitingou in mid-2022 was high, suggesting that COVID-19 cases were asymptomatic or remained undetected by surveillance systems. Despite pregnant women with COVID-19 being at increased risk of pregnancy complications, the proportion of vaccinated participants was lower than the national proportion of the population vaccinated with at least one dose at the time of the survey (23.7%) [49]. Pregnant women should be prioritized for COVID-19 diagnosis and vaccination, in order to prevent deleterious effects of COVID-19 in pregnancy, especially among those with underlying morbidities.

## Data Availability

The datasets used and analysed during the current study are available from the corresponding author on reasonable request.

## Abbreviations

ANC: antenatal care
CI: confidence interval
COVID-19: Coronavirus disease
ELISA: Enzyme-linked immunosorbent assay
MUAC: middle-upper arm circumference
NPV: negative predictive value
OR: odds ratio
PPV: positive predictive value
RDT: rapid diagnostic test
SARS-CoV-2: severe acute respiratory syndrome coronavirus 2
SSA: sub-Saharan Africa
WHO: World Health Organization
